# Superior mesenteric arterial branch occlusion causing partial jejunal ischemia: a case report

**DOI:** 10.1186/1752-1947-6-48

**Published:** 2012-02-06

**Authors:** Nele Van De Winkel, Avine Cheragwandi, Koenraad Nieboer, Franciscus van Tussenbroek, Kristel De Vogelaere, Georges Delvaux

**Affiliations:** 1 Department of Abdominal Surgery, UZ Brussel, Vrije Universiteit Brusse, Brussels, Belgium; 2 Department of Radiology, UZ Brussel, Vrije Universiteit Brusse, Brussels, Belgium

## Abstract

**Introduction:**

Ischemic bowel disease comprises both mesenteric ischemia and colonic ischemia. Mesenteric ischemia can be divided into acute and chronic ischemia. These are two separate entities, each with their specific clinical presentation and diagnostic and therapeutic modalities. However, diagnosis may be difficult due to the vague symptomatology and subtle signs.

**Case presentation:**

We report the case of a 68-year-old Caucasian woman who presented with abdominal discomfort, anorexia, melena and fever. A physical examination revealed left lower quadrant tenderness and an irregular pulse. Computed tomography of her abdomen as well as computed tomography enterography, enteroscopy, angiography and small bowel enteroclysis demonstrated an ischemic jejunal segment caused by occlusion of a branch of the superior mesenteric artery. The ischemic segment was resected and an end-to-end anastomosis was performed. The diagnosis of segmental small bowel ischemia was confirmed by histopathological study.

**Conclusion:**

Mesenteric ischemia is a pathology well-known by surgeons, gastroenterologists and radiologists. Acute and chronic mesenteric ischemia are two separate entities with their own specific clinical presentation, radiological signs and therapeutic modalities. We present the case of a patient with symptoms and signs of chronic mesenteric ischemia despite an acute etiology. To the best of our knowledge, this is the first report presenting a case of acute mesenteric ischemia with segmental superior mesenteric artery occlusion.

## Introduction

Ischemic bowel disease comprises mesenteric ischemia and colonic ischemia. Mesenteric ischemia can be divided into acute and chronic ischemia [1,2]. These are two separate entities, each with their specific clinical presentation and diagnostic and therapeutic modalities. However, diagnosis may be difficult due to the vague symptomatology and subtle signs.

## Case presentation

A 68-year-old Caucasian woman was admitted to our Department of Gastroenterology for persistent abdominal discomfort, melena, anorexia and fever for several months. She had a history of hypertension, cholecystectomy, hysterectomy and bilateral ovariectomy. Further questioning revealed diarrhea, melena and anxiety to eat (sitophobia) due to severe postprandial abdominal pain. She had lost 5 kg of bodyweight prior to presentation.

**Figure 1:**
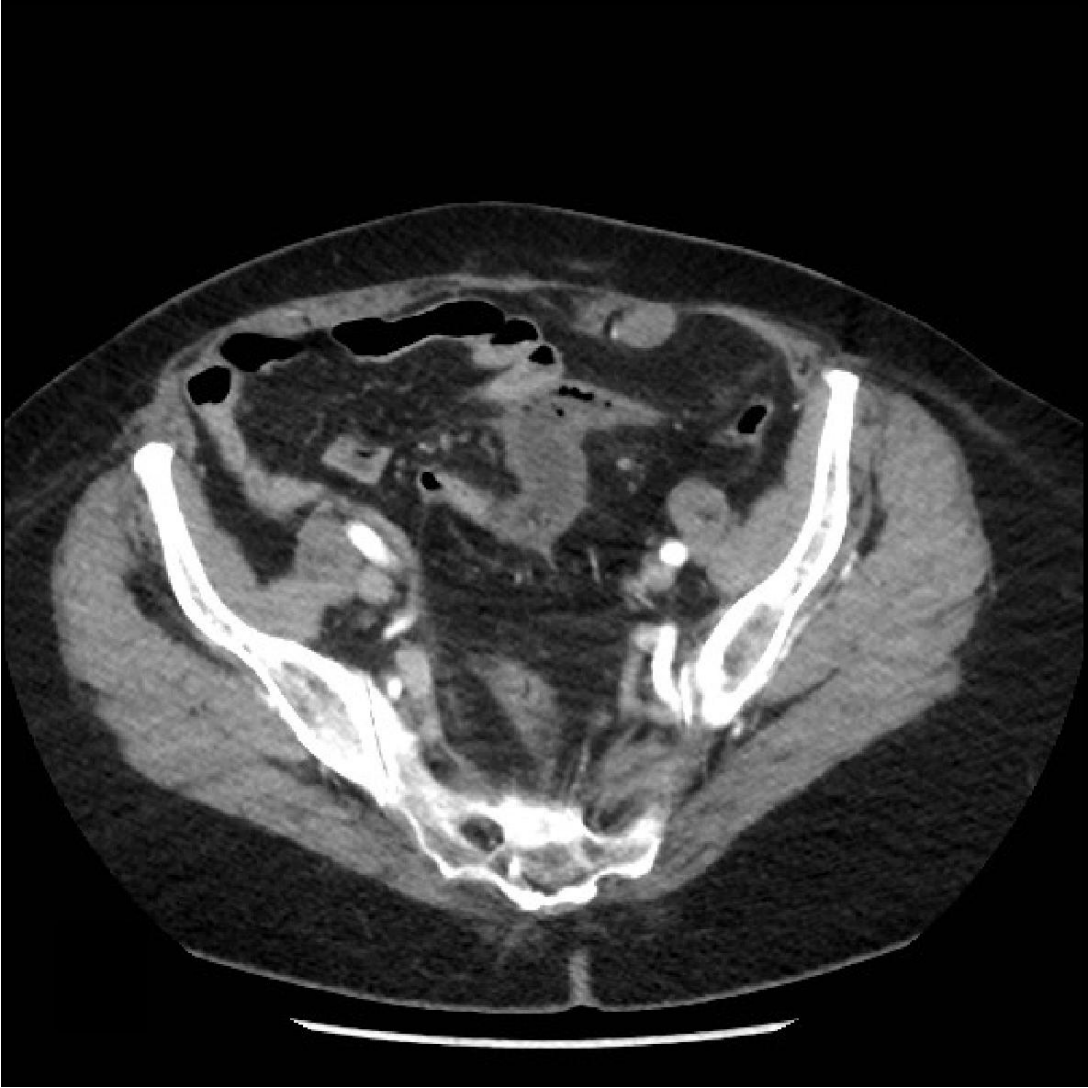
**CT of the abdomen after an injection of iodinated contrast material, presenting absent enhancement of the small bowel wall over a segment**.

**Figure 2:**
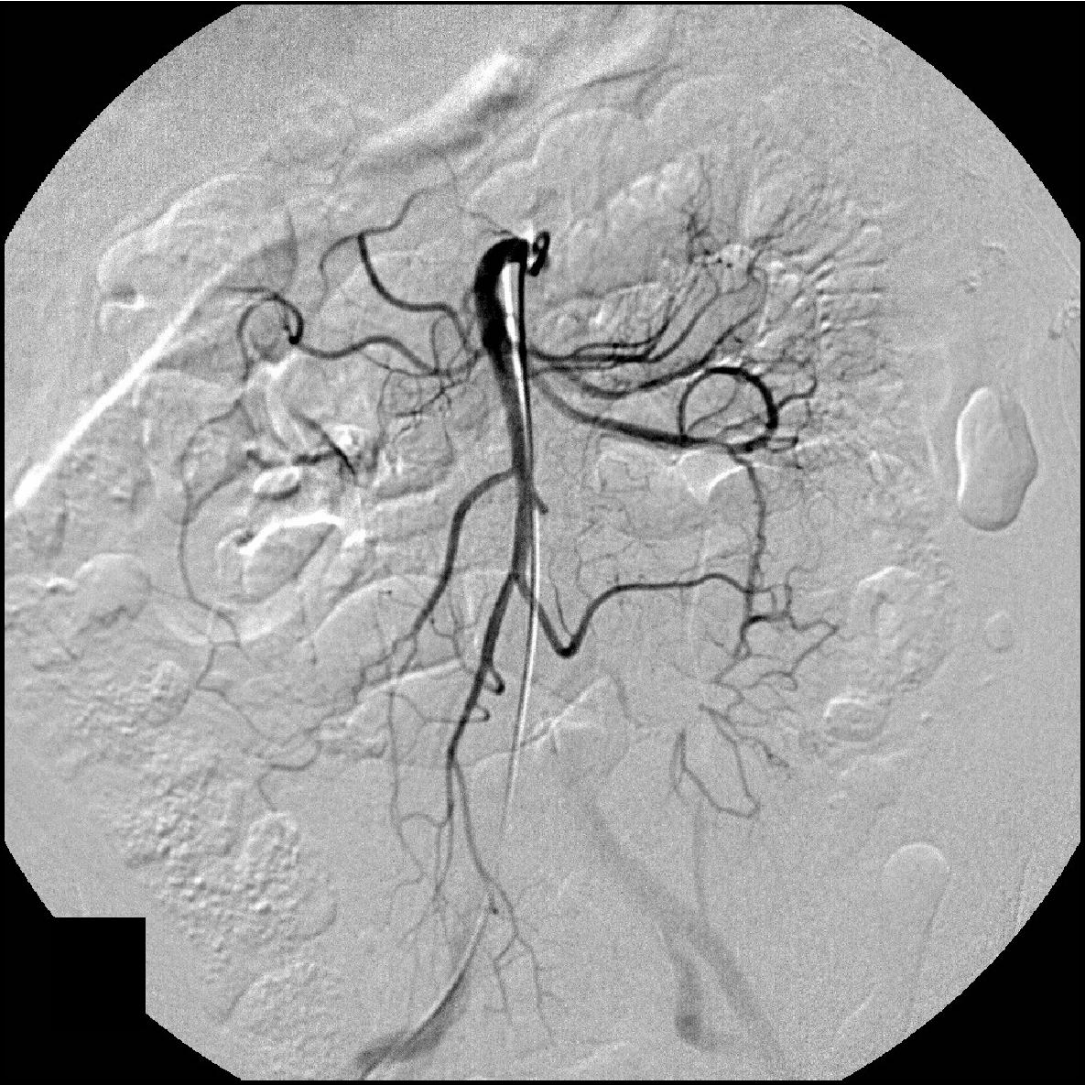
**IADSA demonstrating the solitary occlusion of a branch of the superior mesenteric artery**.

**Figure 3:**
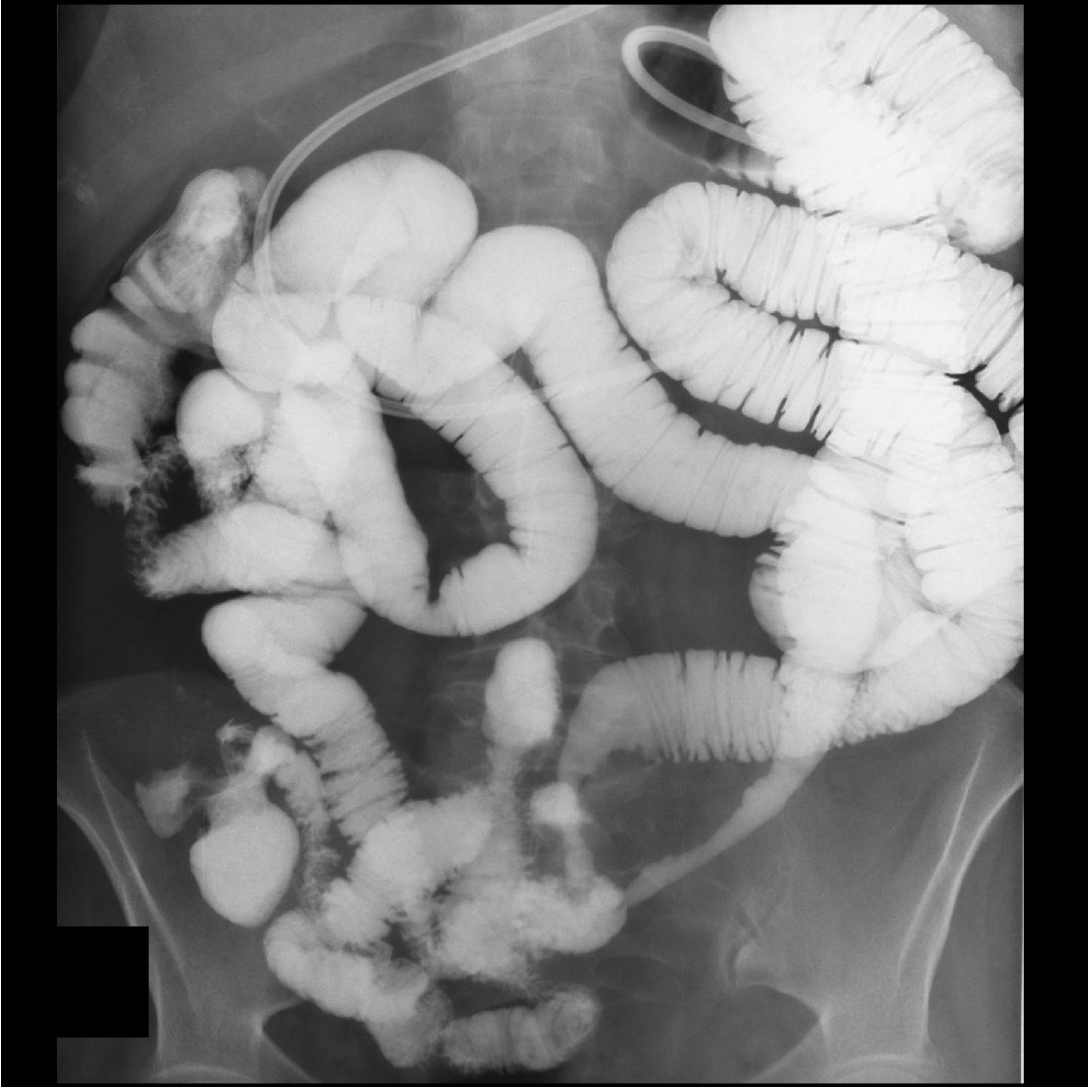
**Enteroclysis, revealing the loss of the normal mucosal pattern of the jejunal segment of the small bowel, suggesting ischemia**.

On physical examination, tender palpation in the left lower quadrant of her abdomen with slight rebound pain was noted. An irregular pulse was also discovered. An electrocardiogram detected paroxysmal atrial fibrillation. Her white blood cell count and C-reactive protein level were elevated. Computed tomography (CT) of her abdomen and CT enterography revealed a stenotic jejunal segment with reduced enhancement of her bowel wall and the presence of inflammation in her left iliac fossa (Figure 1). Enteroscopy showed jejunal ulceration and necrosis one meter distal to the angle of Treitz. Intra-arterial digital subtraction angiography (IADSA) revealed an occlusion of a branch originating from the superior mesenteric artery with collateral vascularization (Figure 2). An enteroclysis confirmed the diagnosis of segmental jejunal ischemia (Figure 3).

**Figure 4:**
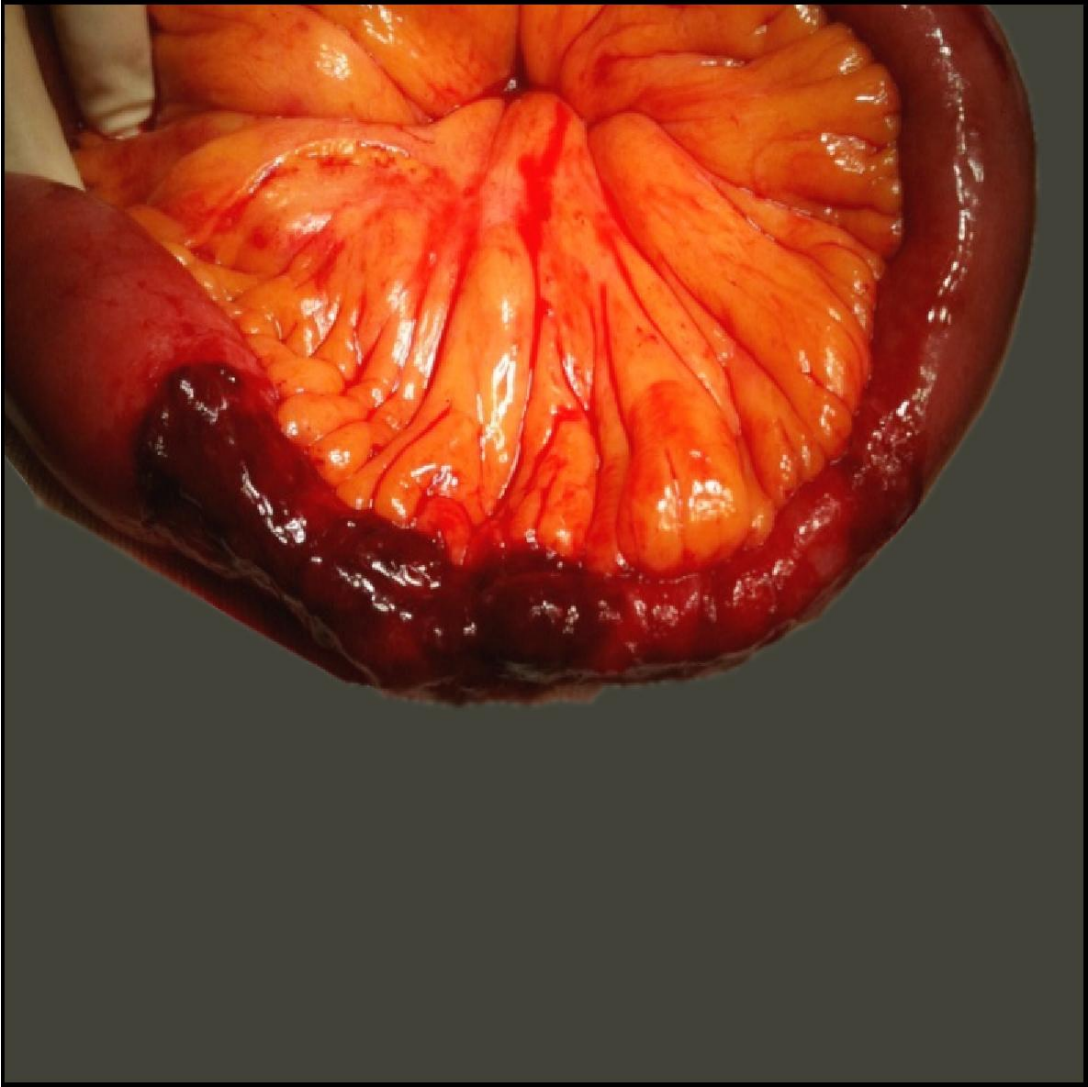
**Perioperative image of the resected ischemic jejunal segment**.

Our patient was transferred to the Department of Abdominal Surgery for exploratory surgery. The stenotic ischemic jejunal segment was resected and an end-to-end anastomosis was performed (Figure 4). A histopathological study revealed a small bowel stricture 10 cm in length, as well as multiple ulcerations with granulation tissue, thickened vascular endothelium and inflammatory infiltrate. These findings corresponded with the clinical and surgical diagnosis of segmental small bowel ischemia.

Our patient recovered from surgery without complications and was discharged on the tenth postoperative day. She underwent radiofrequency ablation for the atrial fibrillation and is currently under anticoagulant therapy. Follow-up has been uneventful to this date.

## Discussion

Acute mesenteric ischemia (AMI) accounts for 60% to 80% of all cases of mesenteric ischemia and has a mortality rate between 59% and 93% [2-7]. AMI can be caused by arterial emboli, arterial or venous thrombosis and non-occlusive obstruction. In all causes, the sudden onset of intestinal hypoperfusion can lead to hypoxemia and intestinal hypoxia with irreversible bowel damage [3,4]. Risk factors for developing AMI depend on the etiology: more often patients are older than 50 years and suffer from congestive heart failure, recent myocardial infarction, hypovolemia, hypotension or sepsis [2-6,8]. Clinical presentation is nonspecific, often presenting as a sudden onset of severe abdominal pain, frequently out of proportion to what is found at physical examination. Accompanying symptoms and signs are nausea, vomiting and hypotension [2,3,5,6,8,9]. The absence of specific signs upon physical examination can make the diagnosis of AMI very challenging and the clinical consequences of missed AMI can be catastrophic. A rapid diagnosis is the most important factor for prognosis, and urgent investigation of vessel patency should thus be done by abdominal CT or IADSA [2,3,5-7,9]. During the work-up, the patient should be closely monitored and stabilized [2]. Once the diagnosis has been made, ischemic bowel segments should be resected and the blood flow restored as soon as possible. Depending on the etiology of the acute ischemia, the restoration of blood flow can be achieved either surgically, using intra-arterial vasodilatation, embolectomy or bypass, or via medical revascularization with, for example, intravenous heparin [2,3,5,8].

Chronic mesenteric ischemia (CMI) is a rare condition that accounts for only 5% of all intestinal ischemic events [1,10,11]. It is caused by atherosclerotic occlusion or severe stenosis of the mesenteric vessels in more than 90% of cases [1-3,6,8-13]. Ischemia occurs when the blood supply to the intestines is inadequate as a result of lesions affecting one or more of the three mesenteric arteries: the celiac artery, the superior mesenteric artery or the inferior mesenteric artery [3,6,10,12]. In most patients, at least two of the three arteries are either completely obstructed or severely narrowed before symptoms of CMI occur [1-3,6-8,10,11,13]. However, CMI due to isolated occlusion of the celiac artery or the superior mesenteric artery has also been described [6,9,12]. Distal lesions carry a greater risk of symptoms than proximal lesions because they are less likely to develop collateral arteries [7,12].

Risk factors for CMI are the same as those for atherosclerosis, namely hyperlipidemia, hypertension, diabetes and smoking [1,2,10,11,13]. Further, most patients are older than 60 years of age, and women are affected three times more frequently than men [2,8-13].

As with AMI, clinical diagnosis is often difficult due to the vague symptomatology. Symptoms of CMI typically develop when there is a postprandial increase in blood flow demand. The most characteristic symptom is intestinal angina. This typical abdominal pain occurs within the first hour after ingestion of a meal. It is often located in the epigastric region or the mid-abdomen and is described by patients as dull and crampy. Diarrhea, nausea and vomiting are commonly present [1,6,8-13]. Most patients have significant weight loss caused by sitophobia [1,6,7,9,10,12]. Physical findings are usually both aspecific and unremarkable and pain is frequently out of proportion to the objective findings on investigation. Sometimes a bruit can be heard over the epigastric region [1,10,11,13].

Duplex ultrasonography, IADSA, CT angiography and magnetic resonance angiography can be used to detect intra-abdominal vascular disease [1,2,6-13]. The treatment goal in CMI is to relieve symptoms, improve nutritional status and prevent progression to acute ischemia [9,10,12,13]. Open surgical revascularization is still considered the first choice for patients with CMI [3,8,13]. However, endovascular procedures are currently considered a viable alternative and are increasingly performed, and the medical literature has confirmed their feasibility and safety with an acceptable rate of patency [2,3,8,10,12,13].

In the current case, confusion existed about the true etiology. There was a discrepancy between her symptomatology, namely intestinal angina, diarrhea and sitophobia suggesting CMI, and the diagnostic sign of an isolated occlusion of a branch of the superior mesenteric artery, which pointed towards the diagnosis of AMI. No reasonable explanation could be found for the symptoms of melena and fever. The AMI was caused by an arterial embolism, most likely due to atrial fibrillation. In this context we may conclude that this case is a late and unusual presentation of AMI. In our opinion, it is the size of the embolism that is responsible for the localization of the ischemic zone. The localization of this zone is a coincidence.

An extensive work-up was done as a consequence of the confusion. An upper gastrointestinal endoscopy and an enteroclysis were performed to indicate the severity, extent and location of the ischemic segment. However they proved unnecessary, as angiography was the most accurate imaging technique [1-13].

Revascularization by operative or endovascular procedures in this case was not possible because of the distal site of the occlusion. Resection of her small bowel segment was the only appropriate action. However, generally, revascularization procedures are mandatory [1,3-13].

As far as we know, this is the first report presenting a case with segmental occlusion of the superior mesenteric artery causing AMI.

## Conclusion

Mesenteric ischemia can be divided into acute and chronic ischemia, two separate entities, each with their own specific clinical presentation and diagnostic and therapeutic modalities. Diagnosis may be difficult due to the vague symptomatology and subtle physical signs. These diagnoses should therefore always be kept in mind in any patient with chronic postprandial abdominal pain in whom no other diagnosis can be made. The diagnosis should be confirmed by angiography. Management of intestinal ischemia consists of blood flow restoration through medical treatment or surgical management, depending on the etiology.

## Consent

Written informed consent was obtained from the patient for publication of this case report and any accompanying images. A copy of the written consent is available for review by the Editor-in-Chief of this journal.

## Competing interests

The authors declare that they have no competing interests.

## Authors' contributions

FvT performed the IADSA and commented together with KN on the radiological images used in the manuscript. AC was a major contributor in writing the manuscript. All authors read and approved the final manuscript.
